# Elevated muscle mass accompanied by transcriptional and nuclear alterations several months following cessation of resistance‐type training in rats

**DOI:** 10.14814/phy2.15476

**Published:** 2022-10-18

**Authors:** Erik P. Rader, Brent A. Baker

**Affiliations:** ^1^ Centers for Disease Control and Prevention National Institute for Occupational Safety and Health Morgantown West Virginia USA

**Keywords:** dorsiflexor muscles, dynamometer, skeletal muscle, stretch‐shortening contractions

## Abstract

Rodent studies investigating long‐term effects following termination of hypertrophy‐inducing loading have predominantly involved exposures such as synergist ablation and weighted wheel running or ladder climbing. This research yielded a spectrum of results regarding the extent of detraining in terms of muscle mass and myonuclei number. The studies were also limited in their lack of sensitive performance measures and indirect relatedness to resistance training. Our research group developed and validated a relevant rat model of resistance‐type training that induces increased muscle mass and performance. The aim of the present study was to determine to what extent these features persist 3 months following the termination of this training. While performance returned to baseline, muscle mass remained elevated by 17% and a shift in distribution to larger muscle fibers persisted. A 16% greater total RNA and heightened mRNA levels of ribosomal protein S6 kinases implicated preserved transcriptional output and ribosomal content. Remodeling of muscle fiber nuclei was consistent with these findings – increased nuclear number and a distribution shift to a more circular nuclear shape. These findings indicate that muscle mass detrains at a slower rate than performance and implicates multiple forms of myonuclear remodeling in muscle memory.

## Graphical abstract

The purpose of the present study was to determine the extent of detraining in a relevant rat model of resistance‐type training. By 3 months following training cessation, performance returned to baseline while muscle mass and transcriptional output was heightened. This was accompanied by alterations in nuclei number and shape thereby supporting the concept of long‐term myonuclear remodeling as a contributor in muscle mass regulation and muscle memory.

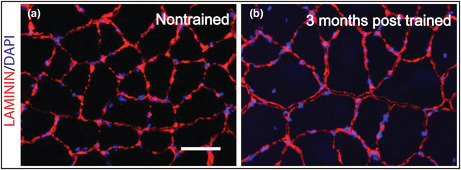

## 1 INTRODUCTION

Loading‐induced hypertrophy with transcriptional upregulation has been observed concomitant with nuclei accretion in various studies regarding both humans and rodents (Abou Sawan et al., 2021; Dungan et al., 2019; Goh et al., 2019; Hammarstrom et al., 2022; Lee et al., 2018; Mesquita et al., 2021; Murach et al., 2020). Bruusgaard et al. in 2010 pioneered work utilizing a model of synergist ablation to cause hypertrophy followed by denervation‐induced atrophy to demonstrate that load‐induced gains in myonuclei could be long‐lasting after the termination of such exposure (Bruusgaard et al., 2010). This finding was consistent with the idea of enduring myonuclear accretion as a form of “muscle memory” allowing enhanced muscle adaptation following a period of detraining.

Subsequent research groups further investigated this possibility in the context of exercise utilizing rodents and various loaded exercise paradigms such as weighted wheel running and ladder climbing (Dungan et al., 2019; Lee et al., 2018; Murach et al., 2020). This research yielded a spectrum of results. During 2 months of weighted wheel running training, both plantaris and soleus muscles increased in myonuclei and muscle mass by 20%–30%, respectively (Dungan et al., 2019; Murach et al., 2020). By 3 months following the cessation of this training, plantaris muscles returned to nontrained values for myonuclei and muscle mass (Dungan et al., 2019). In contrast, soleus muscles did not detrain but rather preserved elevated myonuclei number and muscle mass, even at 6 months following training termination (Murach et al., 2020). In regards to weighted ladder climbing, 2 months following such training, flexor hallucis longus muscles increased muscle mass and myonuclei number by 30% (Lee et al., 2018). By 5 months after ending this training, muscle mass returned to nontrained values, while myonuclei number persisted at trained values (Lee et al., 2018). While these studies were instrumental in highlighting the variation in outcomes possible following load‐induced hypertrophy in general, they were limited in their direct relatedness to resistance training – the predominate form of training utilized to induce hypertrophy. Notably, the weighted wheel running and ladder climbing protocols had high endurance demands and were performed at higher volumes and frequency than resistance‐type training. Furthermore, the studies were limited in their capacity to track sensitive performance changes during and after training.

Our research group has established and repeatedly validated a rat research model to investigate resistance‐type training (Cutlip et al., 2006; Naimo et al., 2019; Rader & Baker, 2017, 2020; Rader, Layner, et al., 2016). The model is based on using a dynamometer to precisely expose dorsiflexor muscles of rats to eight sets of 10 repetitions (per set) of stretch‐shortening contractions (SSCs) – a consecutive sequence of isometric, lengthening, and shortening contractions – at a moderate velocity of 60° ankle rotation per second (i.e., contractions and parameters comparable to those in resistance training for human subjects) (Heckel et al., 2019; Vaczi et al., 2011; Vaczi et al., 2014). Training with this exposure three sessions per week for 1 month results in increases in muscle mass and performance (Cutlip et al., 2006; Naimo et al., 2019; Rader & Baker, 2017). This is accompanied by an increase in muscle fiber area accompanied by a proportional increase in myonuclei number (i.e., myonuclear domain maintenance) and a rise in overall transcriptional output as measured by total RNA levels (Naimo et al., 2019). The purpose of the present study was to determine to what extent alterations in performance, muscle mass, nuclei number, and transcriptional output persist several months following the termination of this relevant and valid model of resistance‐type training. The findings have implications for the variation in the persistence of training‐induced gains in performance, muscle mass, transcriptional output, and myonuclei and the potential role of these features in detraining and muscle memory.

## 2 MATERIALS AND METHODS

### 2.1 Experimental animals

**FIGURE 1:**
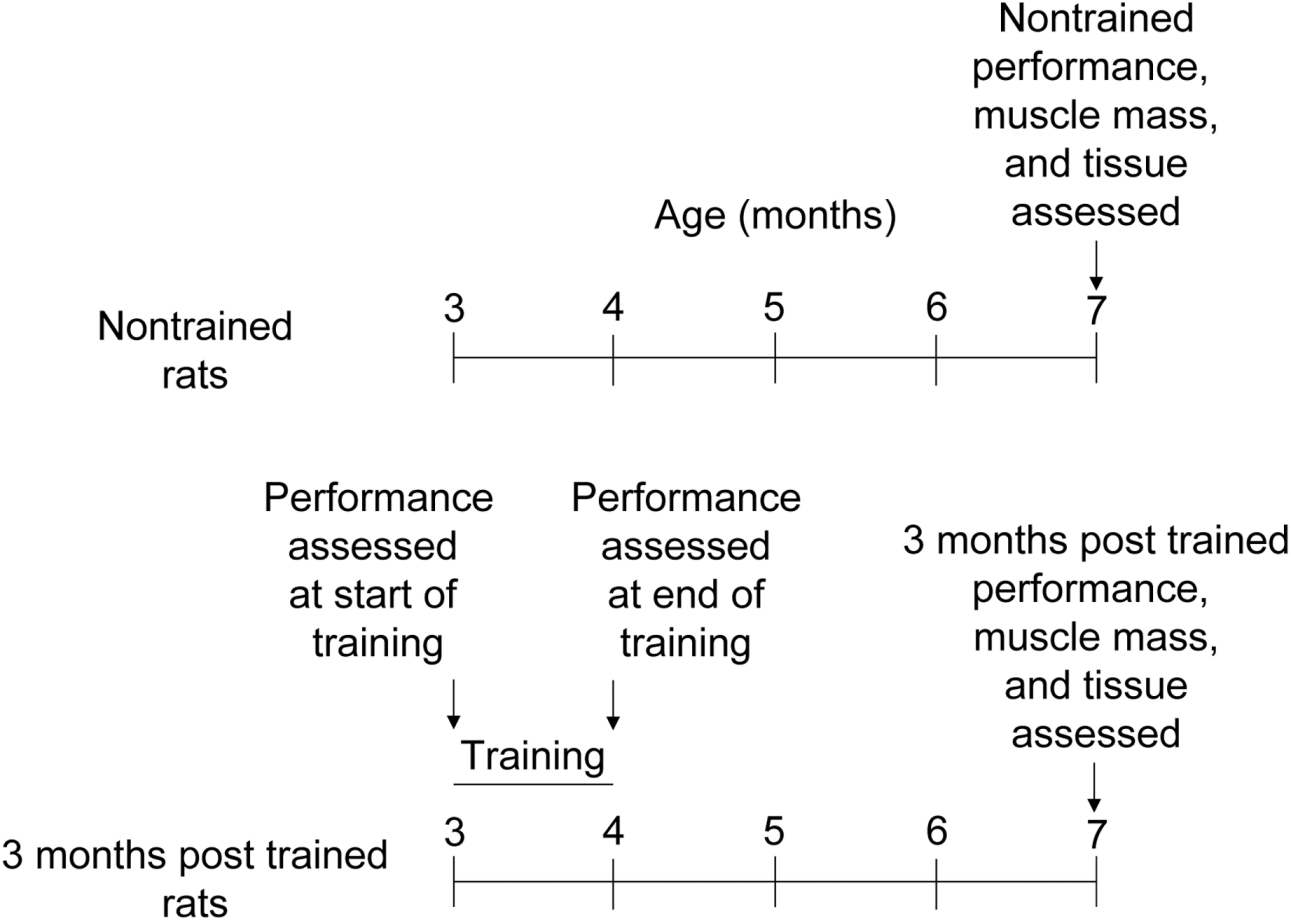
Experimental design. Rats were divided between nontrained and 3 months posttrained groups with 9–10 rats in each group. Training began at 3 months of age and consisted of 3 sessions per week for 4 weeks. For trained rats, performance was assessed at the beginning and end of the 1 month of training and then performance and muscle tissue were evaluated at 7 months of age. For nontrained rats, performance and muscle tissue were assessed exclusively at 7 months of age.

Male Fischer Brown Norway hybrid rats (F344 X BN F1) were obtained from the National Institutes of Aging colony. Rats, 3 months old, were singly housed in an Association for Assessment and Accreditation of Laboratory Animal Care International – accredited animal quarters. Food and water were provided ad libitum. Temperature and light:dark cycle (dark cycle – 7:00 am to 7:00 pm) were held constant. The animals were acclimated to housing conditions for 1 week and randomized to the SSC‐trained group or nontrained group. For the trained group, 3‐month‐old rats were exposed to SSC training for 1 month, thereupon reaching 4 months of age (Figure 1). At 7 months of age, performance testing was completed for both groups of rats – rats that underwent the training and rats which were never trained. After performance testing and while still anesthetized (isoflurane gas 2%–3% by volume), all animals were euthanized by pentobarbital (100–300 mg/kg body weight) intraperitoneal injection followed by exsanguination. All animal procedures were done in accordance with the Guide for the Care and Use of Laboratory Animals (8th edition, National Academies Press) and approved by the Animal Care and Use Committee at the National Institute for Occupational Safety and Health in Morgantown, WV.

### 2.2 SSC training

The SSC training was based on a previously described procedure that resembles high‐intensity resistance training and has repeatedly been demonstrated to induce gains in performance and muscle mass for 3‐month‐old rats (Cutlip et al., 2006; Naimo et al., 2019; Rader & Baker, 2017; Rader, Layner, et al., 2016; Rader, Naimo, et al., 2016). Each rat was anesthetized with isoflurane gas (2%–3% by volume), placed supine on a heated table, the left knee was secured in 90° flexion, and the left foot was secured to a fixture containing a load cell. Platinum electrodes were placed subcutaneously at the region of the common peroneal nerve for activation of dorsiflexor muscles at 4‐V magnitude, 0.2‐ms pulse duration, and 120‐Hz frequency, optimal settings for maximal contraction (Geronilla et al., 2003).

Exposure to SSCs consisted of eight sets with 2‐min intervals between sets and 10 SSCs per set with 2‐sec intervals between SSCs to be comparable to the timing experienced during resistance training (Vaczi et al., 2011). For each SSC, the dorsiflexor muscles were maximally activated, while the ankle was set to 90° for 100 ms (i.e., isometric phase), then rotated to 140° at 60°/s (i.e., stretch phase), returned to 90° at the same velocity (i.e., shorten phase), and lastly, deactivated 300 ms later. The velocity of 60° per second was chosen to promote muscle adaptation rather than overt muscle degeneration and injury – a feature observed at higher velocities (e.g., 500° per second) (Baker et al., 2008; Baker et al., 2010; Cutlip et al., 2006). Each rat was exposed to this protocol three times per week (i.e., Monday, Wednesday, and Friday) for 4 weeks. Performance measures (i.e., torque values for the isometric and stretch phases and work values for the stretch and shorten phases) for the first SSC of each of the sessions during the first and last week of training were averaged to determine initial and final training values, respectively. At 3 months following the cessation of training (i.e., 7 months of age), muscles were exposed to an SSC to assess performance and compared with that of muscles from age‐matched (i.e., 7‐month‐old) nontrained rats. Immediately following this assessment, both right and left tibialis anterior (TA) muscles were surgically removed, weighed, and the tibia lengths recorded.

### 2.3 Total RNA and mRNA analysis

A ~65 mg portion of frozen TA muscle tissue was homogenized with a Mini‐BeadBeater 8 (Biospec) while in a vial of 1 ml of TRIzol with 1.0‐mm zirconia beads (BioSpec, Cat#11079110zx). The RNAqueous phenol‐free total RNA Isolation Kit (Ambion, Cat# AM1912) was used to isolate RNA and total RNA concentration was quantified (NanoDrop 2000c, Thermo Fisher Scientific). The cDNA was synthesized utilizing 0.5 μg of RNA and the RT^2^ First Strand Kit (Qiagen, Cat# 330401). The expression of genes relevant to growth and energy sensing was investigated using the RT^2^ Profiler™ PCR Array for mTOR signaling (Qiagen, Cat# PARN‐098Z) per the manufacturer's instructions. Gene expression was considered significantly differentially regulated when fold regulation exceeded 1.3‐fold regulation (below 0.769‐fold change or above 1.3‐fold change) and *p* < 0.05(Rader et al., 2017; Rader & Baker, 2020).

### 2.4 Immunofluorescence

The mid‐belly of each TA muscle was covered with tissue freezing media and immersed in isopentane (−160°C). This tissue was then cryosectioned at 12 μm thickness. Sections were fixed in HistoChoice (Sigma‐Aldrich; H2904) for 45 min, washed (3 × 5 min in PBS), washed (3 × 5 min in PBS), and then blocked with 5% goat serum in 0.4% Triton X‐100 in PBS for 1 h. A primary polyclonal antibody for laminin (Sigma‐Aldrich; L9393; 1:50) was applied for 1 h. Sections were washed (3 × 5 min in PBS) and secondary antibody (donkey anti‐rabbit IgG Cy3 at 1:100 in PBS with 0.4% Triton X‐100) was applied for 30 min. After 3 × 5 min washes in PBS, sections were mounted with Prolong™ Gold Antifade Reagent (Thermo Fisher Scientific; P36931) with 4′, 6‐diamidino‐2‐phenylindole (DAPI). With the investigator blinded to sample identification, each muscle section was imaged by a standardized method (Baker et al., 2006; Rader et al., 2018; Rader & Baker, 2020; Rader, Naimo, et al., 2016). At 2 mm offset from either side of the midpoint, 5 equally spaced fields (at 20× magnification) were imaged for a total of 10 images. Image analysis utilized ImageJ (version 1.46, National Institutes of Health, USA). Each muscle fiber (118 ± 2 fibers per section) was traced to determine muscle fiber size. Number of nuclei within each muscle fiber was counted to assess number of nuclei per muscle fiber. Nuclei not in contact with the laminin boundary were designated as central nuclei. Muscle fiber area per nucleus was determined for each muscle fiber by dividing the muscle fiber area by the number of nuclei per fiber. ImageJ functions of watershed and particle analysis were utilized for each DAPI image to yield nuclei size and circularity of all the nuclei (2626 ± 70 nuclei per section) in the muscle tissue for the regions imaged (Eidet et al., 2014; Rader & Baker, 2020). Small nuclear fragments (<5 μm^2^) were omitted from the measurements by modifying the particle area range (Eidet et al., 2014). The index of circularity was determined by the equation 4π (area/perimeter^2^) with a perfect circle as a value of 1 and increasing elongation as the value decreases.

### 2.5 Statistical analysis

Data comparing two groups was evaluated by t‐test (with the exception of data regarding central nuclei for which the Mann–Whitney rank sum test was used because normality could not be assumed). Data comparing several groups were analyzed using ANOVA (SigmaPlot version 14.0, Systat Software, Inc) with the variable of animal identification as a random factor to account for repeated measures within animals when appropriate. Post hoc comparisons were performed using Fisher's least significant difference method. Correlation analysis was performed by Pearson product correlation (SigmaPlot version 14.0). Chi‐square analysis (SigmaPlot version 14.0) was utilized to assess differences in the frequency distribution. All data were shown as means ± error in which the error was SD. *p* < 0.05 was considered statistically significant.

## 3 RESULTS

### 3.1 Performance

**FIGURE 2:**
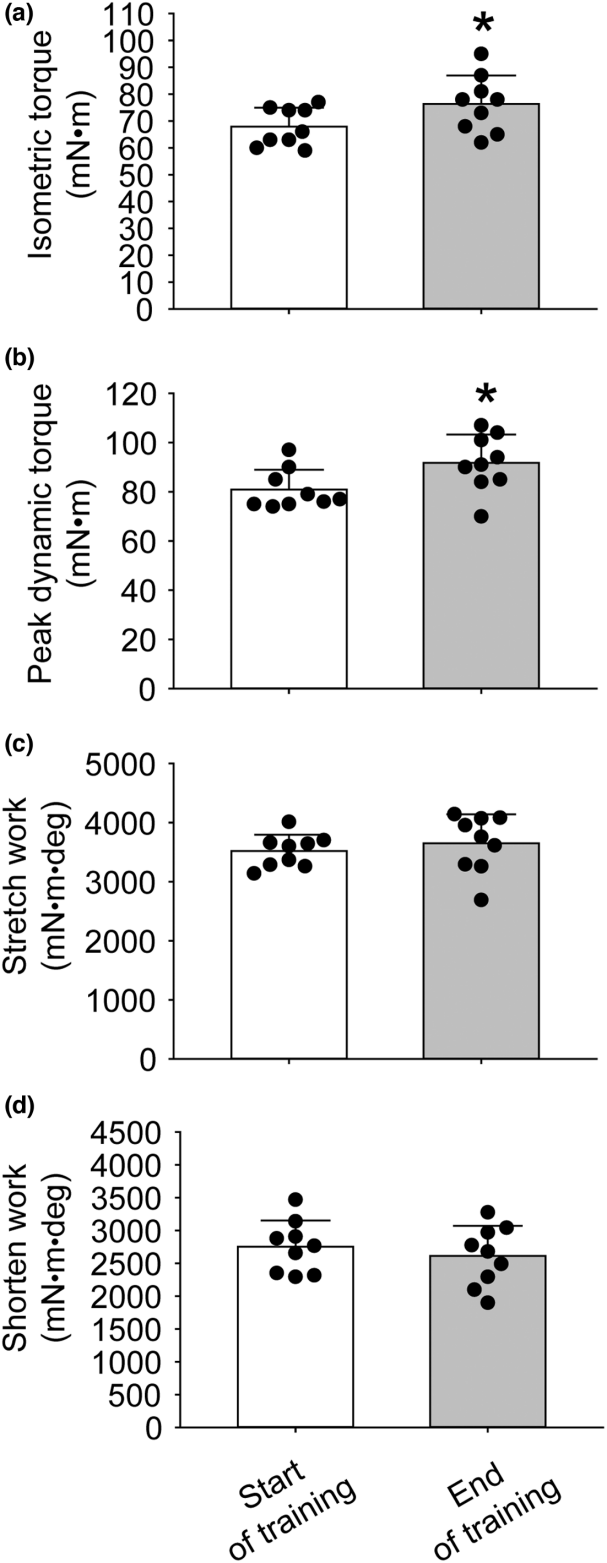
For trained rats, SSC performance measures were assessed initially and at the end of 1 month of training. At 3 months of age, the muscles of rats began training for 3 sessions per week for 1 month. Each session consisted of 8 sets of 10 SSCs with each SSC composed of a static isometric phase immediately followed by stretch and shortening phases. For the first SSC at the beginning and end of the 1 month of training, (a) isometric torque, (b) peak torque during the stretch phase, (c) stretch work, and (d) shorten work were evaluated. The sample size was *N* = 9. Dots represent individual data values. Bar graphs and error bars represent means ± SD. *final value different from initial value, *P* < 0.05.

**FIGURE 3:**
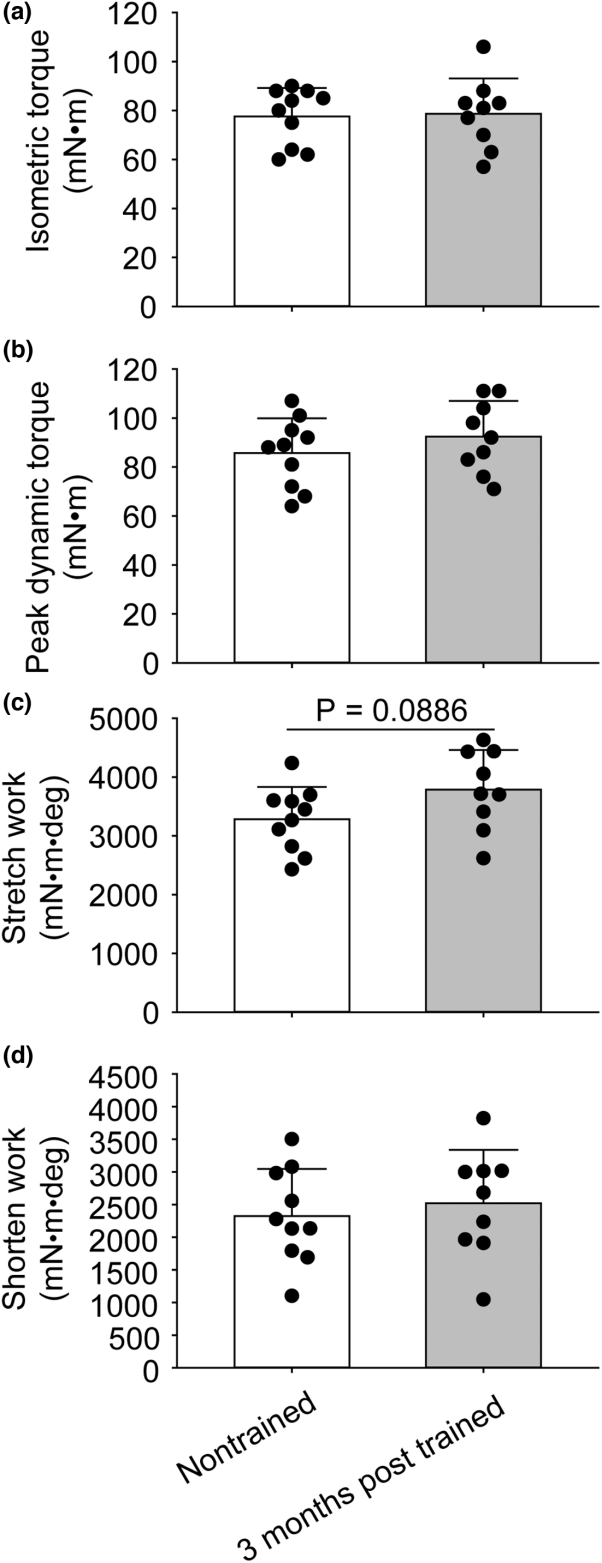
SSC performance measures for muscles of rats 3 months after training was terminated and muscles of rats which were nontrained. For the trained group, the muscles of rats 3 months of age were exposed to SSC sessions 3 days per week for 1 month thereby reaching 4 months of age. For the nontrained group, muscles were not exposed to training SSCs. At 7 months old, SSC performance was evaluated by assessing the isometric torque of the initial static phase of an SSC (a), peak torque during the stretch phase (b), work during the stretch phase (c), and work during the shortening phase (d). The sample size was *N* = 9–10 per group. Dots represent individual data values. Bar graphs and error bars represent means ± SD. No significant differences were observed.

Consistent with prior studies utilizing the same SSC training, static and dynamic maximal performance was enhanced during training (Figure 2). Isometric torque and peak dynamic torque increased by 12% and 14%, respectively (Figure 2a,b). By 3 months after training cessation, a pattern of a greater work capacity during stretching was observed when compared with that of nontrained muscles, but significance was not reached, and no other performance differences were observed (Figure 3).

### 3.2 Muscle mass

**FIGURE 4:**
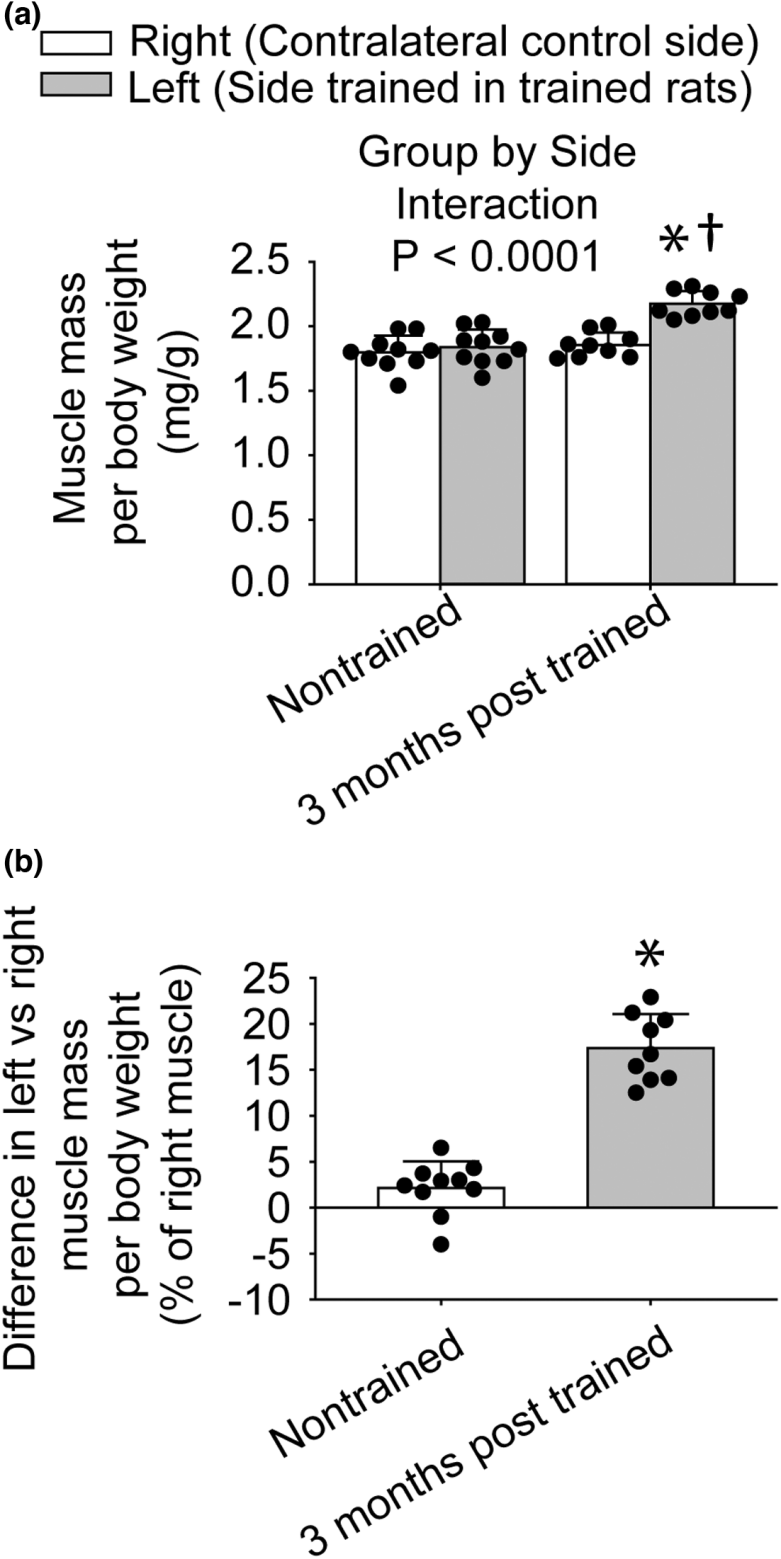
Increased muscle mass persisted 3 months following training. For trained rats, left muscles were exposed to the training while right muscles were not. For nontrained rats, both left and right muscles were not trained. Muscle mass assessment was performed by normalizing muscle mass to body weight (a) and expressing the difference between left and right normalized muscle masses as a percentage of right normalized muscle mass (b). The sample size was *N* = 9–10 per group. Dots represent individual data values. Bar graphs and error bars represent means ± SD. *value for right muscles distinct from left value, ^†^value for 3 months posttrained different from the value for nontrained, *p* < 0.05.

A greater muscle mass of 17% was observed 3 months posttraining relative to nontrained (Figure 4). For muscle mass normalized to body weight, a group (3 months posttrained vs. nontrained) by side (left vs. right muscles) ANOVA interaction was observed for muscle mass normalized to body weight (Figure 4a). Left muscles were trained in the 3 months posttrained group and right muscles were not trained for any of the groups. The interaction of group‐by‐side was also observed for absolute muscle mass (793 ± 38 mg vs. 677 ± 49 mg; left vs. right of 3 months posttrained and 718 ± 42 mg vs. 703 ± 31 mg; left vs. right of nontrained) and muscle mass normalized to tibia length (18.7 ± 0.9 mg/mm vs. 16.0 ± 1.1 mg/mm; left vs. right of 3 months posttrained and 16.8 ± 1.0 mg/mm vs. 16.5 ± 0.7 mg/mm; left vs. right of nontrained) (*p* < 0.05). No difference in muscle quality (isometric torque divided by muscle mass normalized to tibia length, values all available for the left side) was observed between 3 months posttrained (4.18 ± 0.67 mN•m/mg/mm) and nontrained muscles (4.65 ± 0.81 mN•m/mg/mm), *p* = 0.195.

### 3.3 Total RNA and mRNA

**FIGURE 5:**
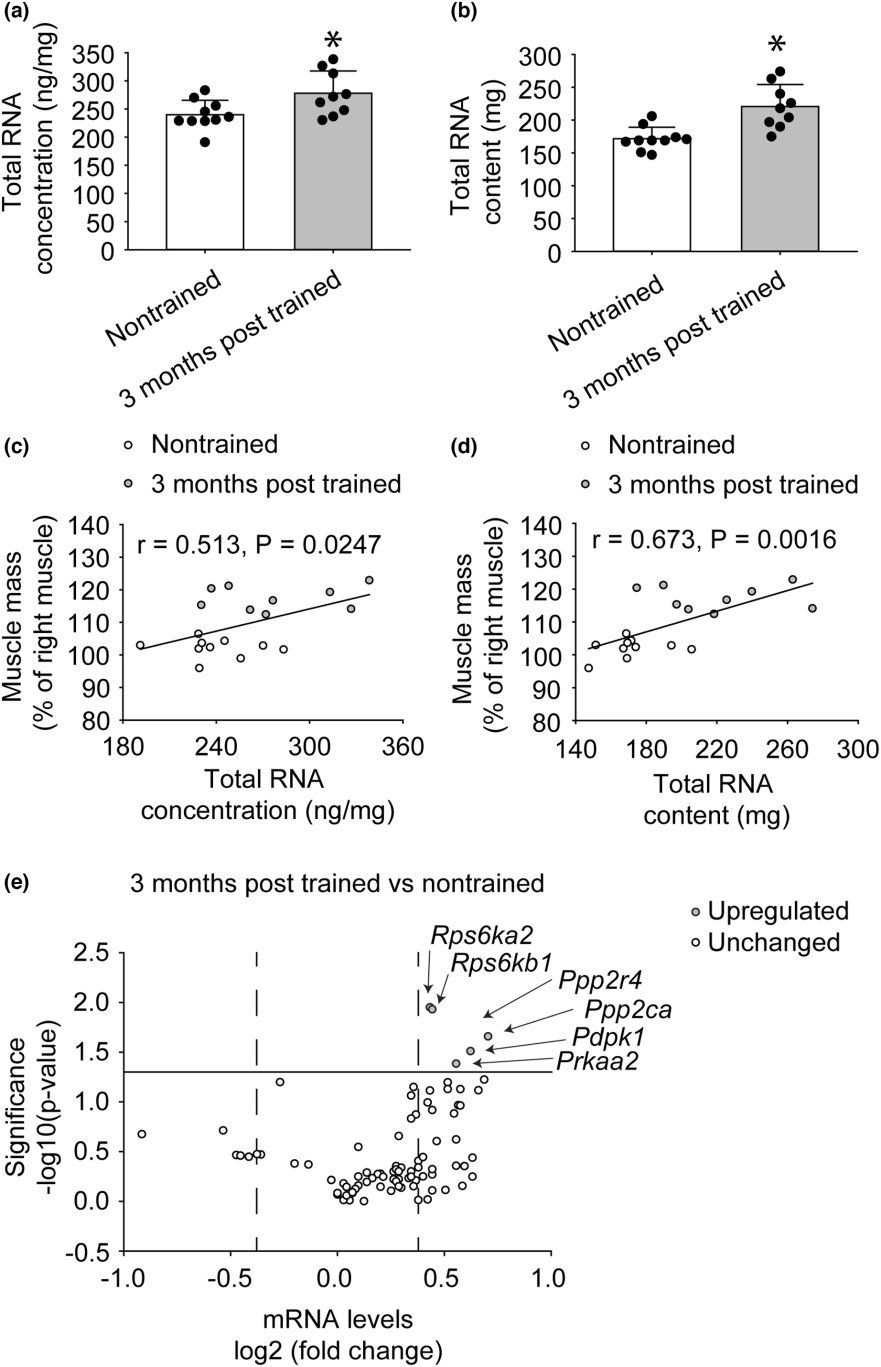
Heightened response at the transcriptional level accompanied the increased muscle mass 3 months posttraining. Greater total RNA and mRNA levels for genes relevant to growth and energy sensing – ribosomal protein S6 kinases, serine/threonine phosphatases, and a catalytic subunit of AMPK – were observed. Total RNA was evaluated in terms of total RNA concentration (ng/mg tissue) (a) and content (mg) per muscle (b). Dots represent individual data values. Bar graphs and error bars represent means ± SD. *value for 3‐month posttrained different from value for nontrained, *p* < 0.05. Muscle mass correlated with both total RNA concentration (c) and total RNA content (d). Values represent individual data points. A PCR array for gene expression was utilized to evaluate differential mRNA levels of 3‐month posttrained vs. nontrained rats. Refer to table S1 for more detailed information. The solid line denoted a *p* value cut‐off of 0.05 and the dashed lines represented 1.3‐fold regulation. The mRNA which surpassed these thresholds was identified. The sample size was *N* = 9–10 per group.

To investigate whether a more pronounced response at the transcriptional level accompanied the greater muscle mass several months posttraining, total RNA was analyzed. Total RNA concentration was higher by 16% for 3‐month posttrained relative to nontrained muscle and correlated with muscle mass (Figure 5a,b). Likewise, total RNA content per muscle was greater by 28% posttraining versus nontrained and demonstrated a correlation with muscle mass (Figure 5c,d). At 3 months posttraining, upregulation of mRNA levels of multiple genes relevant to growth and energy‐sensing were observed (Figure 5e and Table 1). Upregulation of genes for ribosomal protein S6 kinases (*Rps6ka2* and *Rps6kb1*), 3‐phosphoinositide‐dependent protein kinase 1 (*Pdpk1*), serine/threonine phosphatases (*Ppp2r4* and *Ppp2ca*), and a catalytic subunit of AMPK (*Prkaa2*) were observed.

**TABLE 1:** Differential mRNA levels between 3 months post trained and nontrained muscles

	RefSeq	3 months post trained vs. nontrained			RefSeq	3 months post trained vs. nontrained	
		Fold change	*p* value			Fold change	*p* value
*Akt1*	NM_033230	1.21	0.443259	*Pik3r2*	NM_022185	1.55	0.565163
*Akt1s1*	NM_001106259	1.35	0.076905	*Pld1*	NM_030992	1.15	0.529263
*Akt2*	NM_017093	1.07	0.695075	*Pld2*	NM_033299	1.27	0.565748
*Akt3*	NM_031575	1.05	0.827537	*Ppp2ca*	NM_017039	1.63	0.021939
*Cab39*	NM_001106924	1.10	0.514909	*Ppp2r2b*	NM_022209	0.72	0.343008
*Cab39l*	NM_001011917	1.04	0.978551	*Ppp2r4*	NM_001108577	1.55	0.017368
*Rps6ka4*	NM_001108517	1.36	0.777937	*Prkaa1*	NM_019142	1.29	0.134019
*Cdc42*	NM_171994	1.02	0.973250	*Prkaa2*	NM_023991	1.47	0.041255
*Chuk*	NM_001107588	1.21	0.488901	*Prkab1*	NM_031976	1.34	0.958219
*Ddit4*	NM_080906	1.27	0.500980	*Prkab2*	NM_022627	1.49	0.074509
*Ddit4l2*	NM_080399	0.91	0.427323	*Prkag1*	NM_013010	1.43	0.063573
*Eif4b*	NM_001008324	1.29	0.617896	*Prkag2*	NM_184051	1.00	0.827929
*Eif4e*	NM_053974	1.20	0.503042	*Prkag3*	NM_001106921	1.14	0.532881
*Eif4ebp1*	NM_053857	1.03	0.875194	*Prkca*	NM_001105713	1.43	0.074247
*Eif4ebp2*	NM_001033069	1.48	0.107941	*Prkcb*	NM_012713	0.77	0.336371
*Fkbp1a*	NM_013102	1.23	0.459309	*Prkce*	NM_017171	1.36	0.475564
*Fkbp8*	NM_001037180	1.19	0.786289	*Prkcg*	NM_012628	0.73	0.347938
*Gsk3b*	NM_032080	1.58	0.076384	*Pten*	NM_031606	1.27	0.086244
*Hif1a*	NM_024359	1.21	0.478434	*Rheb*	NM_013216	1.22	0.220807
*Hras*	NM_001098241	1.00	0.855454	*Rhoa*	NM_057132	1.16	0.567477
*Hspa4*	NM_153629	1.30	0.393348	*Rps6*	NM_017160	1.09	0.997158
*Igf1*	NM_178866	0.87	0.418021	*Rps6ka1*	NM_031107	1.07	0.564559
*Igfbp3*	NM_012588	1.22	0.600981	*Rps6ka2*	NM_057128	1.35	0.011184
*Ikbkb*	NM_053355	1.28	0.706371	*Rps6ka5*	NM_001108048	1.50	0.700878
*Ilk*	NM_133409	1.49	0.108660	*Rps6kb1*	NM_031985	1.36	0.011717
*Ins2*	NM_019130	0.53	0.211391	*Rps6kb2*	NM_001010962	1.34	0.101258
*Insr*	NM_017071	1.47	0.439700	*Rptor*	NM_001134499	1.23	0.730543
*Irs1*	NM_012969	1.36	0.538165	*Rraga*	NM_053973	0.75	0.356836
*Kras*	NM_031515	1.32	0.359479	*Rragb*	NM_053972	1.21	0.631648
*Mapk1*	NM_053842	1.06	0.752397	*Rragc*	NM_001048184	1.27	0.146956
*Mapk3*	NM_017347	1.15	0.714059	*Rragd*	NM_001106641	1.12	0.585126
*Mapkap1*	NM_001011964	1.46	0.131018	*Sgk1*	NM_019232	1.28	0.070840
*Mlst8*	NM_022404	1.30	0.972572	*Stk11*	NM_001108069	1.36	0.120784
*Mtor*	NM_019906	1.30	0.456898	*Tp53*	NM_030989	0.69	0.193737
*Myo1c*	NM_023092	1.47	0.239220	*Tsc1*	NM_021854	1.22	0.708038
*Nras*	NM_080766	1.22	0.500642	*Tsc2*	NM_012680	1.05	0.812045
*Pdpk1*	NM_031081	1.54	0.030863	*Ulk1*	NM_001108341	1.32	0.562445
*Pik3c3*	NM_022958	1.26	0.585449	*Vegfa*	NM_031836	1.51	0.442975
*Pik3ca*	NM_133399	1.61	0.059696	*Vegfb*	NM_053549	1.42	0.770054
*Pik3cb*	NM_053481	1.20	0.600632	*Vegfc*	NM_053653	0.61	0.304077
*Pik3cd*	NM_001108978	1.38	0.248681	*Ywhaq*	NM_013053	1.02	0.662148
*Pik3cg*	NM_001106723	0.78	0.339246	*Rplp1*	NM_001007604	0.98	0.611012
*Pik3r1*	NM_013005	1.55	0.364853				

*Note*: Expression which surpassed 1.3‐fold regulation (below 0.77‐fold change or above 1.3‐fold change) with a *p* value <0.05 was considered differentially expressed. Not highlighted – unchanged, orange – upregulated. Sample sizes were *N* = 9–10 per group.

### 3.4 Muscle fiber and nuclei analysis in transverse sections

**FIGURE 6:**
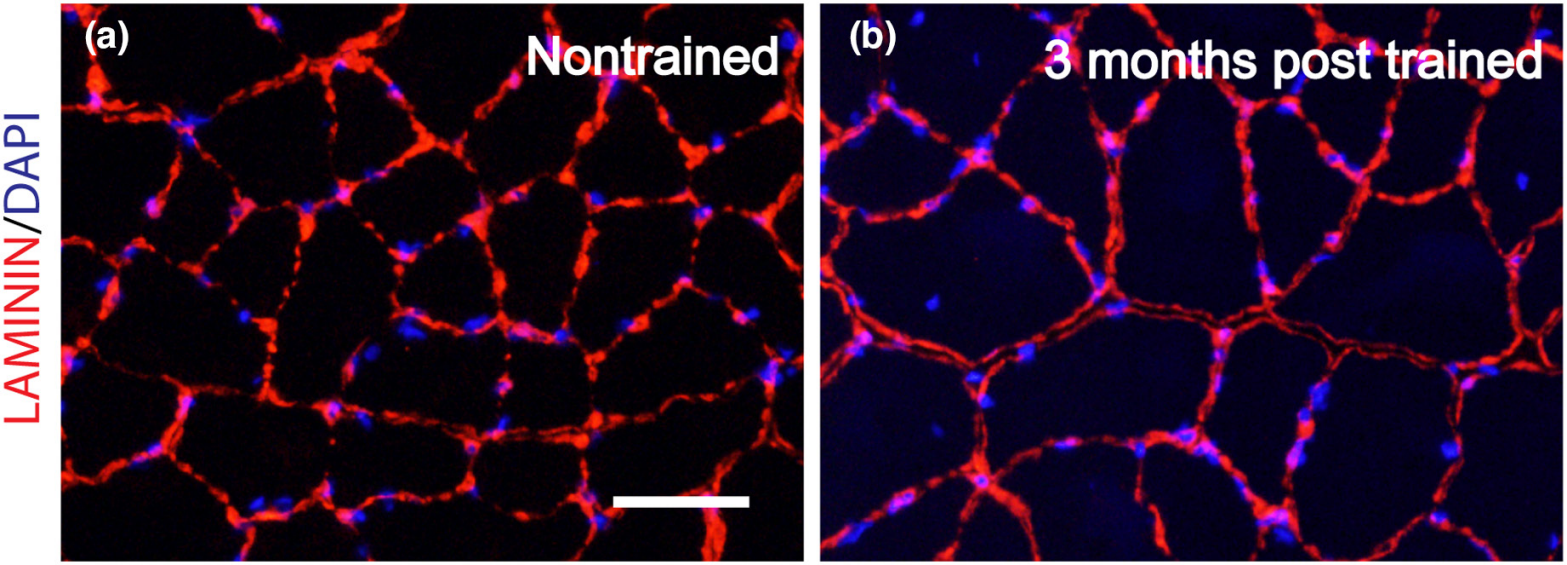
Transverse sections of nontrained (a) and 3 months posttrained (b) muscles of rats were labeled by immunofluorescence for laminin (red) and nuclei (blue). Scale bar = 50 μm.

**FIGURE 7:**
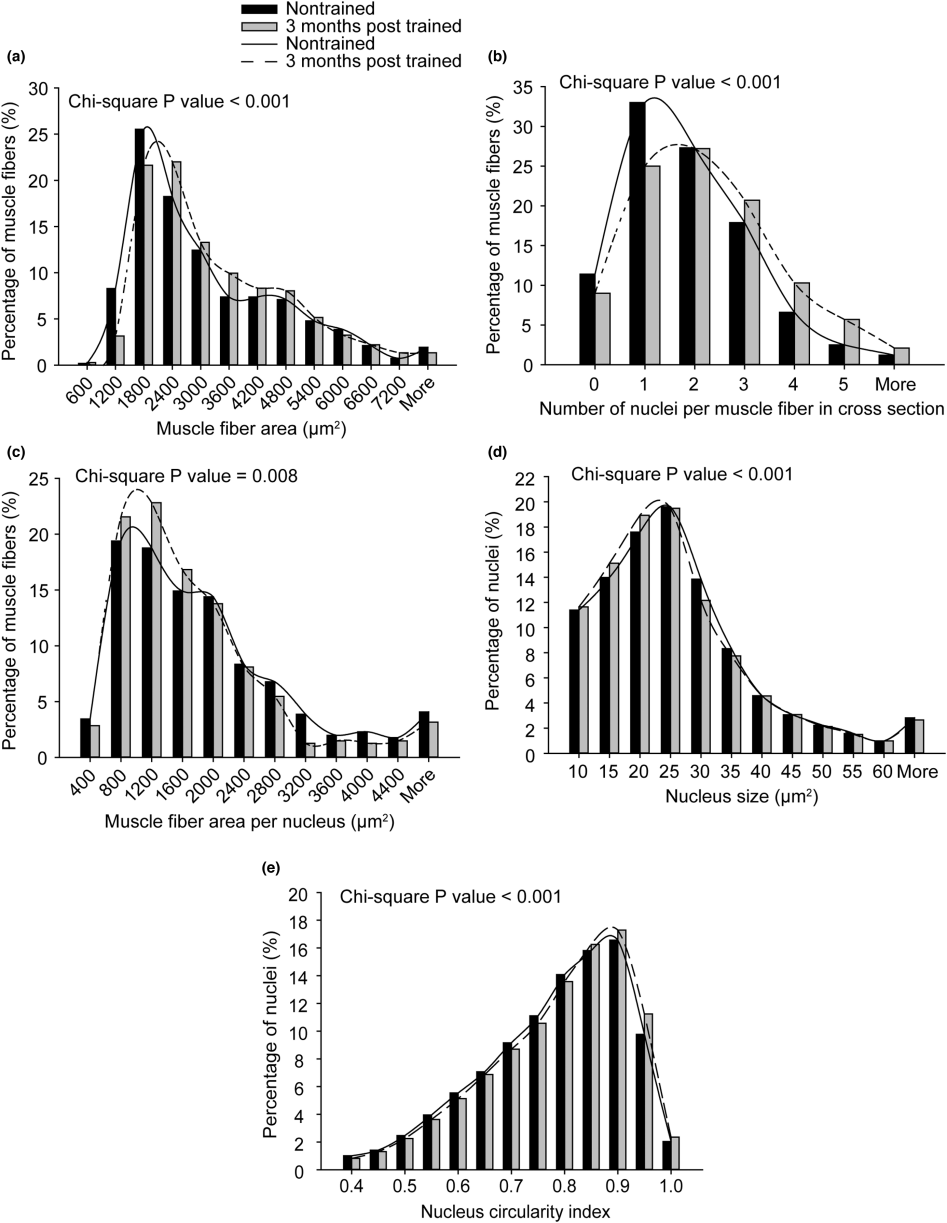
Distribution shifts to larger muscle fibers coincided with alterations in nuclei – increased nuclei number per muscle fiber and a greater extent of nuclei circularity. Frequency distributions of muscle fiber area (a), number of nuclei per muscle fiber (b), muscle fiber area per nucleus (c), nucleus size (d), and nucleus circularity index (e) for nuclei of muscle tissue were determined for both groups (*N* = 9 muscles evaluated per group). The nucleus circularity index denotes a perfect circle at a value of 1 and increasing elongation at lower values. Chi‐square analysis was performed to evaluate alterations in distributions between groups, *p* < 0.05.

To examine muscle fiber and nucleus morphology concomitant with the enhanced transcriptional response and muscle mass gain 3 months posttraining, muscle sections were analyzed by immunofluorescence labeling for laminin and nuclei (Figure 6a,b). Evidence of remodeling was apparent by a trend for a greater percentage of central nucleated fibers (11.2 ± 3.1% for 3 months posttrained vs. 7.0 ± 7.2% for nontrained, *p* = 0.052) and a significant difference in central nuclei per unit area of muscle tissue (43 ± 14 per mm^2^ vs. 29 ± 40 per mm^2^ for nontrained, *p* = 0.034). Regarding the additional morphological measurements assessed, when mean data were analyzed, significant differences were not reached (e.g., 3 months posttrained vs. nontrained – muscle fiber area: 2992 ± 277 μm^2^ vs. 2845 ± 212 μm^2^, number of nuclei per muscle fiber: 2.2 ± 0.6 vs. 1.9 ± 0.3, muscle fiber area per nucleus: 1553 ± 304 μm^2^ vs. 1702 ± 174 μm^2^, nucleus size: 20.3 ± 2.1 μm^2^ vs. 20.5 ± 1.0 μm^2^, and nucleus circularity index: 0.782 ± 0.008 vs. 0.777 ± 0.015, *p* > 0.05). However, when the entire distribution of data for each measure was accounted for and analyzed by chi‐square analysis, significant differences emerged (Figure 7). Consistent with the muscle mass increase, a shift to larger muscle fibers was observed 3 months posttraining (Figure 7a). This was accompanied by an even greater shift to more nuclei per muscle fiber, thereby translating to a general shift to smaller myonuclear domains (Figure 7b,c). Analysis of nucleus morphology demonstrated a shift to a smaller nucleus size (Figure 7d). This was concomitant with a shift to more circular nuclei (Figure 7e).

## 4 DISCUSSION

The present study demonstrated in a relevant and valid rat model of resistance‐type training that several months following cessation of such training, muscle mass gains persisted (and to a greater extent than performance gains) and were correlated with transcriptional output. Furthermore, these outcomes were present in the context of alterations at the level of the nuclei – increased nuclei number and greater nuclear circularity, both features which were consistent with heightened transcriptional capacity and muscle mass. These findings support the notions that muscle mass detrains at a slower rate than performance, and that candidate nuclear alterations operative in muscle memory should extend beyond solely examining nuclei number – namely, nuclei shape should be considered as an additional mechanism of muscle memory.

Research regarding humans is incomplete in regards to characterizing the extent of detraining for performance versus muscle size (Bosquet et al., 2013; Coratella et al., 2021; Mujika & Padilla, 2001; Staron et al., 1991). Our study supports the notion that changes in performance are more rapid upon training/detraining than that for muscle size. Previous reports from our research group repeatedly reported greater increases in performance measures relative to those in muscle mass during 1 month of the same resistance‐type training of the present study (Cutlip et al., 2006; Naimo et al., 2019; Rader & Baker, 2017). Given this enhanced adaptation in performance during training, the absence of any heightened isometric or dynamic torque 3 months after training terminated (while a 17% muscle mass gain persisted) was striking and highlighted the relative resilience of muscle size (i.e., muscle mass and muscle fiber area). The shift in distribution to larger muscle fibers relative to nontrained muscle fibers was consistent with muscle fiber hypertrophy contributing to the retained muscle mass. This finding suggests that the lack of heightened performance in the presence of greater muscle mass was due to factors (e.g., neuromuscular junction, excitation‐contraction coupling, myofibrillar packing, etc.) other than simply an absence of muscle fiber hypertrophy. This relative persistence of muscle mass gain has been noted before for the soleus muscle 6 months following a weighted wheel running rodent model of hypertrophy (Murach et al., 2020). In that study, the researchers proposed that the chronic activation of the soleus as a weight‐bearing muscle contributed to the maintenance of hypertrophy. Our finding of a long‐lasting muscle mass increase in the TA muscle demonstrated this phenomenon was possible in a nonweight bearing muscle following the resistance‐type training of the present study.

Total RNA levels were increased 3 months posttraining and correlated with muscle mass outcomes in the present study. This increase in general transcriptional output was accompanied by heightened mRNA levels for specific genes relevant to growth and energy sensing – ribosomal protein S6 kinases, serine/threonine phosphatases, and a catalytic subunit of AMPK. While there are certain circumstances when growth signaling (such as the mechanistic target of rapamycin complex 1 signaling, mTORC1) and energy sensing AMPK signaling appear to compete (e.g., following endurance exercise for trained individuals), increases in both signaling responses can coexist – for example, following resistance‐type exercise in previously untrained individuals (akin to the present study) (Mesquita et al., 2021). Heightened levels of ribosomal protein S6 kinases such as *Rps6kb1* in the present study were interesting given the role as a key mTORC1 substrate and inducer of ribosome biogenesis (Marabita et al., 2016). Increased total RNA was also suggestive of ribosomal biogenesis since the majority of total RNA (>85%) is ribosomal RNA (Figueiredo & McCarthy, 2019). Other shorter‐term studies regarding humans and rats have observed an increase in total RNA and ribosome biogenesis during multiple sessions of isometric muscle training (Hammarstrom et al., 2022; Kotani et al., 2019; Kotani et al., 2021). The total RNA data of the present study suggest that heightened transcriptional output and ribosomal content persist several months after resistance‐type training; and thereby provide an underlying rationale for the preservation of long‐term muscle mass.

Alterations in nuclei number and shape have the potential to impact transcriptional output (Mascetti et al., 2001; Murach et al., 2022; Rader & Baker, 2020; Versaevel et al., 2012). Muscle fiber size generally scales with myonuclear number in both rodents and humans during growth and load‐induced hypertrophy (Abou Sawan et al., 2021; Goh et al., 2019; Hansson et al., 2020). A previous study also demonstrated that weighted wheel running training in mice induces an influx of satellite cell‐derived myonuclei which contribute to the ribosomal pool of muscle fibers during hypertrophy (Murach et al., 2022). Besides myonuclear number, an alternative type of remodeling at the level of the nucleus is shape change resulting from mechanosensing at the nucleus and signaling (e.g., transcriptional coactivator peroxisome‐proliferator‐activated receptor‐γ coactivator 1‐α) (Ross et al., 2017; Versaevel et al., 2012). Adaptation to a more circular (i.e., less elongated) shape has the potential to decondense chromatin and thereby promote overall transcription (Mascetti et al., 2001; Rader & Baker, 2020; Versaevel et al., 2012). During weighted wheel running training in mice, the myonuclear number increases, the myonuclear shape becomes less elongated, and muscle mass increased for both gastrocnemius and soleus muscles (Murach et al., 2020). Six months following the termination of such training, myonuclear number/shape and muscle mass returned to baseline for gastrocnemius muscles, while these features retained some degree of their trained state for the frequently active weight‐bearing soleus muscle (Murach et al., 2020). The present study was notable in demonstrating that long‐lasting alterations in nuclei number and morphology accompany muscle mass gain in the non‐weight bearing tibialis anterior muscle following resistance‐type training. The persistent nature of these alterations in the present study provides additional impetus to investigate myonuclear remodeling (morphology as well as myonuclear domain) in future work regarding muscle memory for human participants at a variety of ages.

## AUTHOR CONTRIBUTIONS

EPR and BAB conceived and designed the study, performed the experiments/collected data, analyzed and interpreted the data, and drafted and revised the manuscript. All authors read and approved the final manuscript.

## CONFLICT OF INTEREST

The authors declare there are no competing interests.

## ETHICS STATEMENT

All animal experimental procedures performed in this study were approved by the Animal Care and Use Committee at the National Institute for Occupational Safety and Health in Morgantown, WV.
